# Optimizing Frozen Sample Preparation for Laser Microdissection: Assessment of CryoJane Tape-Transfer System®

**DOI:** 10.1371/journal.pone.0066854

**Published:** 2013-06-21

**Authors:** Yelena G. Golubeva, Roberta M. Smith, Lawrence R. Sternberg

**Affiliations:** Pathology-Histotechnology Laboratory, Science Applications International Corporation-Frederick, Frederick National Laboratory for Cancer Research, Frederick, Maryland, United States of America; National Cancer Institute, National Institutes of Health, United States of America

## Abstract

Laser microdissection is an invaluable tool in medical research that facilitates collecting specific cell populations for molecular analysis. Diversity of research targets (e.g., cancerous and precancerous lesions in clinical and animal research, cell pellets, rodent embryos, etc.) and varied scientific objectives, however, present challenges toward establishing standard laser microdissection protocols. Sample preparation is crucial for quality RNA, DNA and protein retrieval, where it often determines the feasibility of a laser microdissection project. The majority of microdissection studies in clinical and animal model research are conducted on frozen tissues containing native nucleic acids, unmodified by fixation. However, the variable morphological quality of frozen sections from tissues containing fat, collagen or delicate cell structures can limit or prevent successful harvest of the desired cell population via laser dissection. The CryoJane Tape-Transfer System®, a commercial device that improves cryosectioning outcomes on glass slides has been reported superior for slide preparation and isolation of high quality osteocyte RNA (frozen bone) during laser dissection. Considering the reported advantages of CryoJane for laser dissection on glass slides, we asked whether the system could also work with the plastic membrane slides used by UV laser based microdissection instruments, as these are better suited for collection of larger target areas. In an attempt to optimize laser microdissection slide preparation for tissues of different RNA stability and cryosectioning difficulty, we evaluated the CryoJane system for use with both glass (laser capture microdissection) and membrane (laser cutting microdissection) slides. We have established a sample preparation protocol for glass and membrane slides including manual coating of membrane slides with CryoJane solutions, cryosectioning, slide staining and dissection procedure, lysis and RNA extraction that facilitated efficient dissection and high quality RNA retrieval from CryoJane preparations. CryoJane technology therefore has the potential to facilitate standardization of laser microdissection slide preparation from frozen tissues.

## Introduction

Laser microdissection (LM) of frozen tissue sections has been successfully used for genomic and proteomic studies in clinical and research laboratories [1]–[3]. LM projects requiring RNA retrieval are especially challenging due to the unstable nature of RNA. Stability of RNA, morphological appearance of the target cells on the dissecting screen, and adherence of sections to the glass or membrane slides varies significantly for different tissues. A customized, target dependent approach to all steps of LM (sample preparation, dissection and nucleic acid/protein retrieval and analysis) has proven to be the most reliable for successful LM [2], [4]–[7].

Frozen sections are considered optimal for genomic studies [2], [5], [7], [8], but a range of tissues containing collagen, fat, cartilage, bone and delicate structures (e.g. mouse normal ovarian epithelium) as well as plant tissues are not suitable for routine serial cryosectioning. For such tissues, random loss of material during cryotomy and staining, as well as poor section morphology is common [9].

The CryoJane Tape-Transfer System® (CryoJane) (Leica Microsystems, GmbH, Nussloch, Germany) was developed by Instrumedics Inc. (Richmond, IL, USA) to improve the outcome of cryosectioning, which requires a high degree of technical skill to acquire intact sections. The system is installed inside the cryostat and equilibrated to the cryosectioning temperature. Cryosectioning is then performed with the help of a thin adhesive tape applied to the frozen tissue block with a hand roller. The frozen section is captured on the prechilled tape during cutting. The tape with the attached section is then rolled onto an adhesive glass slide, that, when subjected briefly to irradiation by ultra-violet light, results in polymerization of slide adhesive into a plastic layer. This firmly anchors the frozen section to the glass slide; the tape is then peeled of the section.

The glass CryoJane slides have been used successfully in both plant and bone research including LM projects [10]–[12]. Considering that the CryoJane procedure aids in successful serial sectioning of a frozen tissue block and in protecting tissue RNA due to the low temperature (−25–30°C) of cryotomy, we evaluated CryoJane for the optimization of LM sample preparation for frozen tissues. We adapted CryoJane for both infra-red laser capture microdissection (IR-LM) performed on glass or glass slides with mounted polyethylene naphthalate membrane (PEN slides) and for ultra-violet laser cutting microdissection (UV-LM) performed on metal frame mounted polyethylene terephthalate membrane (PET slides) and PEN slides. The ability to use CryoJane on different types of LM slides allows flexibility of LM project design and standardization of LM sample preparation.

The goal of our study was to determine the suitability of the CryoJane technique as a standard method of LM slide preparation from frozen tissues. We tested the CryoJane for use with different types of lasers to evaluate its applicability to complex LM projects involving tissues with different RNA stability, and tissues difficult for serial sectioning by routine cryotomy.

## Materials and Methods

### Samples

To assure control of conditions during tissue collection, and minimize sample preanalytical variability, mouse tissues (instead of clinical human samples) with high, medium and low RNA stability (liver, ovary and skin, respectively [4]) were tested. Mouse tissues were collected under conditions approved by the Frederick National Laboratory for Cancer Research, an AAALAC accredited institution that follows the Public Health Service Policy for the Care and Use of Laboratory Animals outlined in the “Guide for Care and Use of Laboratory Animals” [13]; Frederick National Laboratory for Cancer Research ACUC 10-249 M3 approval on 9/20/2012. Mouse liver, skin and ovaries were embedded in Tissue-Tek® OCT Compound (OCT) (Sakura Finetek USA, Inc., Torrance, CA, USA) on a mixture of dry ice and 2-methylbutane (Fisher Scientific, Fair Lawn, NJ, USA) within five minutes of animal euthanasia. Frozen blocks were stored at −70°C prior to cryotomy and handled on dry ice.

### Cryotomy

**Figure 1:**
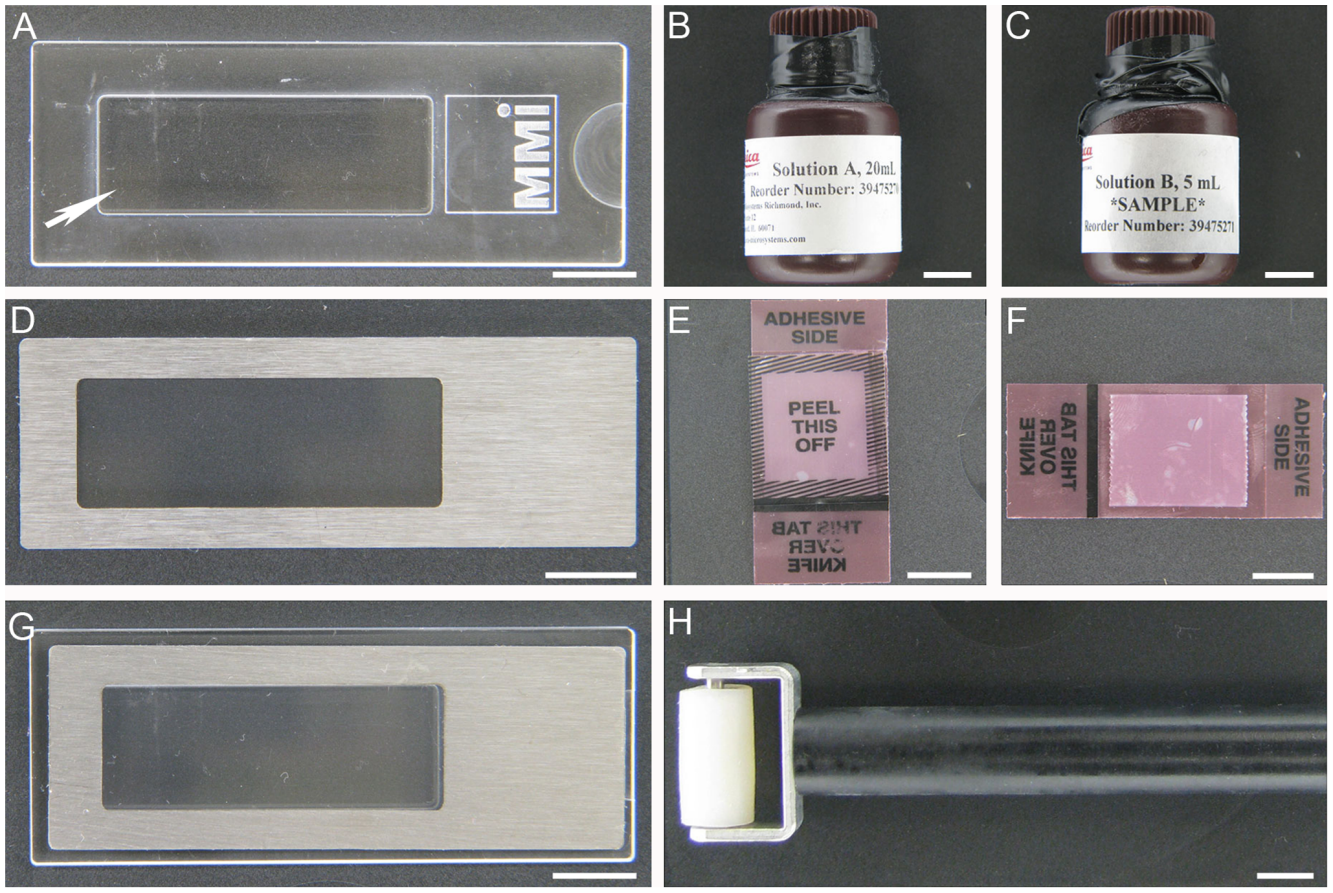
CryoJane technique consumables for metal frame PET membrane slides. (A) MMI SupportSlide: arrow indicates a solid plastic platform which snaps into the well window of the membrane slide; (B, C) CryoJane Solution A and Solution B, respectively; (D) metal frame PET membrane slide; (E, F) Simulation of Transfer Adhesive Tape application: (E) “PEEL THIS OFF” window rolled on the OCT block, (F) Tape with the attached cryosection after cryotomy; (G) PET slide-SupportSlide assembly prior to transfer of CryoJane section to the membrane; (H) CryoJane Hand Roller for application of CryoJane section to the membrane. Scale bars correspond to 1000 µm.

**Figure 2:**
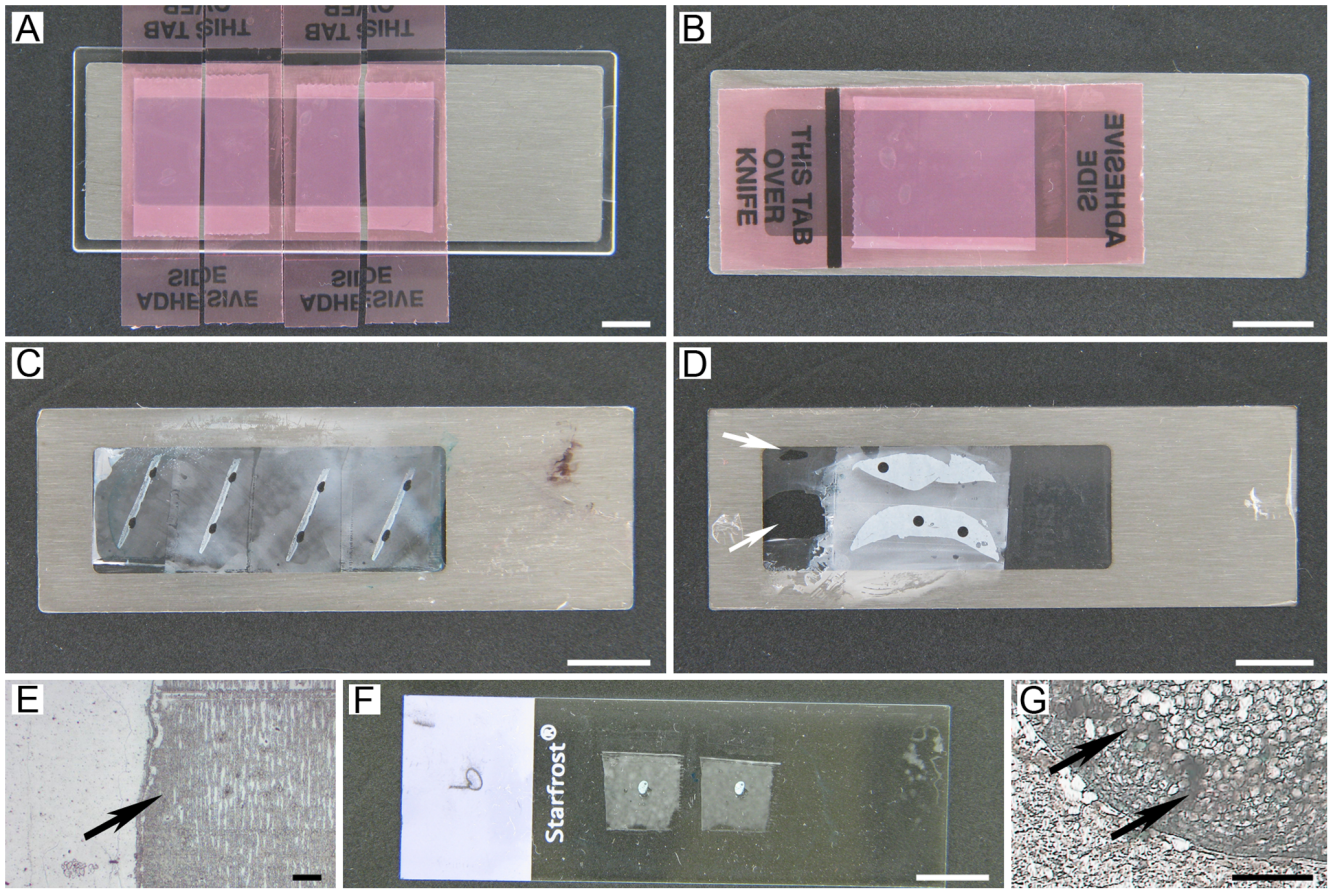
Section mounting on glass-CJ and PET-CJ slides for laser capture (IR-LM) and laser cutting (UV-LM). (A, C) Demonstration of correct mounting position of Adhesive Tape with OCT over slide window, and membrane view following correct mounting and UV-LM of targets from serial sections, respectively; (B, D) Demonstration of incorrect mounting position of Adhesive Tape: (B) Exposed adhesive around the section in the “PEEL THIS OFF” area of the tape touches the membrane; (D) Membrane view following incorrect section mounting and UV-LM of targets (arrows indicate damage); (E) View of adhesive (arrow) on the membrane after staining (MMI CellCut Plus, 4x); (F) Correct tissue block size and mounting position of serial sections on glass-CJ slide for IR-LM; (G) View of ovarian section on glass-CJ slide after staining (arrows indicate OCT media deposits (PixCell® IIe, 10x)). A–D, F: Scale bars correspond to 1000 µm; E, G: Scale bars correspond to 100 µm.

A Leica CM 3050S (Leica Microsystems, GmbH, Nussloch, Germany) cryostat with installed CryoJane, was used for cryosectioning. Frozen 10 µm tissue sections were mounted onto ½ × type commercial CryoJane slides (glass-CJ slides), or metal frame PET membrane slides (MMI Molecular Machines & Industries, Glattbrugg, Switzerland) manually coated with CryoJane solutions (PET-CJ slides) according to the CryoJane manufacturer’s instructions. A plastic MMI SupportSlide (MMI Molecular Machines & Industries, Glattbrugg, Switzerland) was used to facilitate section mounting on PET-CJ slides with a CryoJane Hand Roller (Fig. 1A, D, G, H). Glass++slides (Thermo Fisher Scientific, Waltham, MA, USA) with routine cryosections of mouse ovary and regular PET slides with liver and skin sections served as a control for tissue morphology, laser focusing, target pick-up/laser cutting efficiency and RNA quality in dissected LM targets, and liver sample was used for RNA quality assessment of coating conditions. To speed up dissection and assure maximum RNA amount and quality in the extracted sample, we mounted only the number of sections that could be dissected within a time frame established for each tissue (Fig. 2C, F).The Transfer Adhesive Tape (Leica Microsystems, GmbH, Nussloch, Germany) was cut to the width of a trimmed tissue block or the size of the tissue (Fig. 2A, F). Sections were cut using the cryostat “automated function” for thickness consistency which is important for LM cutting efficiency.

RNAse-free conditions were maintained throughout the procedure as previously described [4], including (a) RNaseAWAY™ (Molecular BioProducts, San Diego, CA, USA) to wipe all the surfaces and tools except the cryostat chamber, (b) 100% ethanol (AAPER Alcohol and Chemical Co., Shelbyville, KY, USA) to wipe the cryostat chamber, (c) a new blade for each block, (d) trimming and discarding 20 µm of tissue from the face of the block at the start of cryotomy. The cryostat object holder and chamber were kept at −24°C and −29°C, respectively. Following sectioning, each mounted slide was immediately moved to a Styrofoam box with dry ice. Slides were then stored in plastic slide boxes sealed in plastic bags at −70°C for 2 weeks prior to LM.

### Fixation and Staining

Fixation and staining were carried out in 50 ml conical polypropylene tubes (Falcon, Franklin Lakes, NJ, USA) containing 30 ml of solution for glass-CJ slides or 45 ml for PET-CJ slides. RNAse-free conditions were maintained throughout the procedure as described previously [4].

Mounted slides were moved (while on dry ice) from a slide box into acetone which was prechilled on dry ice for 1 hour. The slides were fixed in acetone for 15 seconds and then post-fixed in 100% ethanol with 3% glacial acetic acid (Mallinckrodf Baker Inc., Phillipsburg, NJ, USA) for 1 minute at 0°C (on wet ice). Sections were stained with Vector®Methyl Green (Vector Laboratories, Inc., Burlingame, CA, USA) mixed with ProtectRNA™RNAse inhibitor (1∶500) (Sigma, Saint Louis, MO, USA) to protect RNA during LM staining [14]. One milliliter of stain was applied to sections twice for 20 seconds, draining the slides on Kimwipes between applications. The slides were dehydrated in two changes of 100% ethanol for 30 seconds, and cleared in two changes of xylene (EMD Chemicals, Inc. Cincinnati, OH) for 5 minutes. After 5 minutes of air drying in the hood, slides were ready for LM. Three slides per each sample were kept in a desiccator for 15 minutes prior to LM to evaluate the effect of additional drying on laser focusing and laser cutting efficiency.

Stained slides were digitally imaged with an Aperio Scan Scope®XT scanner according to the manufacturer’s directions. The quality of staining and morphological details was then evaluated with Image Scope™ software.

### Coating of PET Slides with CryoJane Solutions

The Adhesive “Solution B” (adhesive) and the Solution A (pretreatment solution) (Leica Microsystems, Richmond, IL, USA) (Fig. 1B, C) were tested for manual coating of PET slides used for UV-LM.

The following coating protocols were tested sequentially: 1) 20, 30 or 50 µl of adhesive was applied to a membrane without incubation in acetone based pretreatment solution to avoid membrane detachment from the metal frame, known to happen during slide incubation in solvents; 2) 10, 15, 30 or 35 µl of adhesive was applied to the slides after they were dipped 4 times in pretreatment solution, slowly removed at an angle, checked for membrane detachment, dried overnight in the hood, and stored in a new slide box at room temperature (RT) prior to adhesive application.

ApopTag® Plastic coverslips (Millipore, Billerica, MA, USA) cut to the height of the PET slide window were soaked for 5 minutes in RNAseAWAY™, then rinsed in RNase-free water and air dried between two layers of Kimwipes at RT. Application of adhesive was performed in a fume hood in dim light to prevent premature polymerization. A drop of adhesive was pipetted on the slide membrane and lightly pressed with the edge of a plastic coverslip. While maintaining the coverslip pressure, the slide was slowly pulled in the direction opposite to the coverslip to ensure even distribution of adhesive inside the membrane window of PET slide. Each coated slide was immediately transferred to a Flip Top Lid, 5 Place Slide Mailer (Globe Scientific Inc., Paramus, New Jersey, U.S.A.) protected from light by aluminum foil. Slides were stored at RT prior to cryotomy on the same day.

### Laser Microdissection

**Figure 3:**
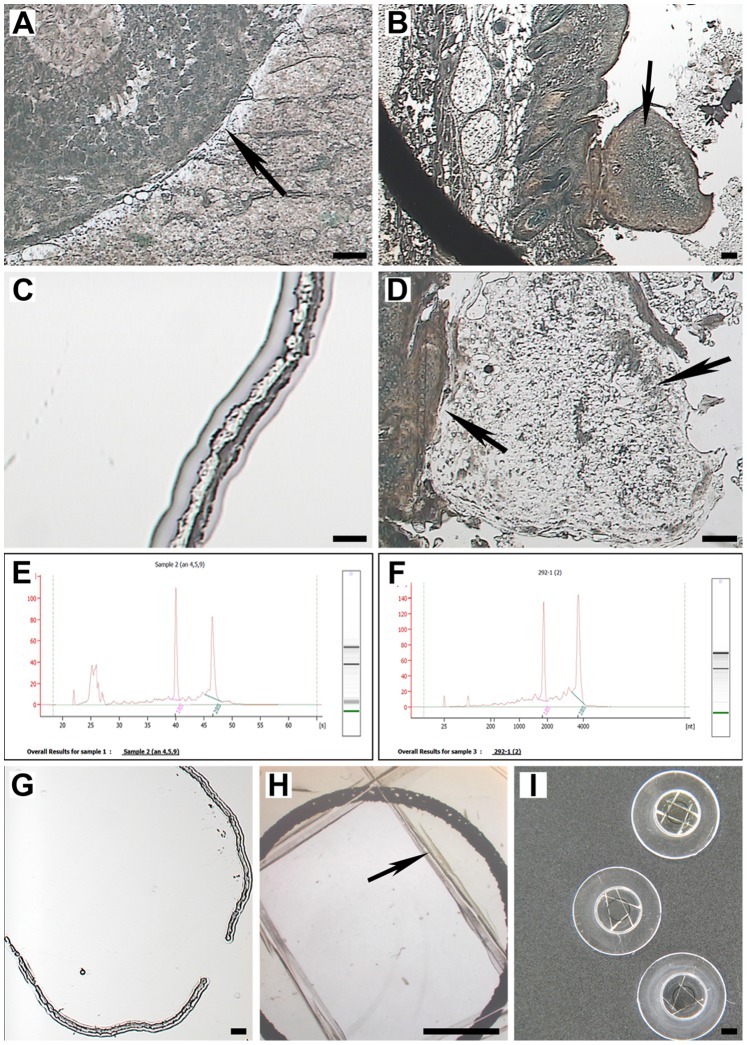
IR-LM on glass-CJ slides (Methyl Green stain) and quality of RNA retrieved from LM targets. (A) IR-LM of mouse normal ovarian epithelium: arrow indicates the target area after removal of captured tissue (PixCell® IIe, 10x); (B) IR-LM of mouse skin (PixCell® IIe, 4x): arrow indicates LM target-papilloma; (C) Captured ovarian epithelium on HS LCM Cap (PixCell® IIe, 20x); (D) View of adhesive left on a slide after removal of captured papilloma (PixCell® IIe, 10x): left arrow indicates the papilloma base and right arrow indicates adhesive; (E, F) Representative Agilent electropherogram of high quality LM RNA extracted from mouse ovarian epithelium and skin papilloma, respectively; (G, H) Captured target on HS LCM Cap (PixCell® IIe, 4x), and a view of LCM Cap film after removal of target cutout, respectively: arrow indicates film remaining on the cap; (I) View of HS LCM Caps after removal of the film areas with embedded target. A, B, D, G: Scale bars correspond to 100 µm; C: Scale bar corresponds to 25 µm; H, I: Scale bars correspond to 1000 µm.

**Figure 4:**
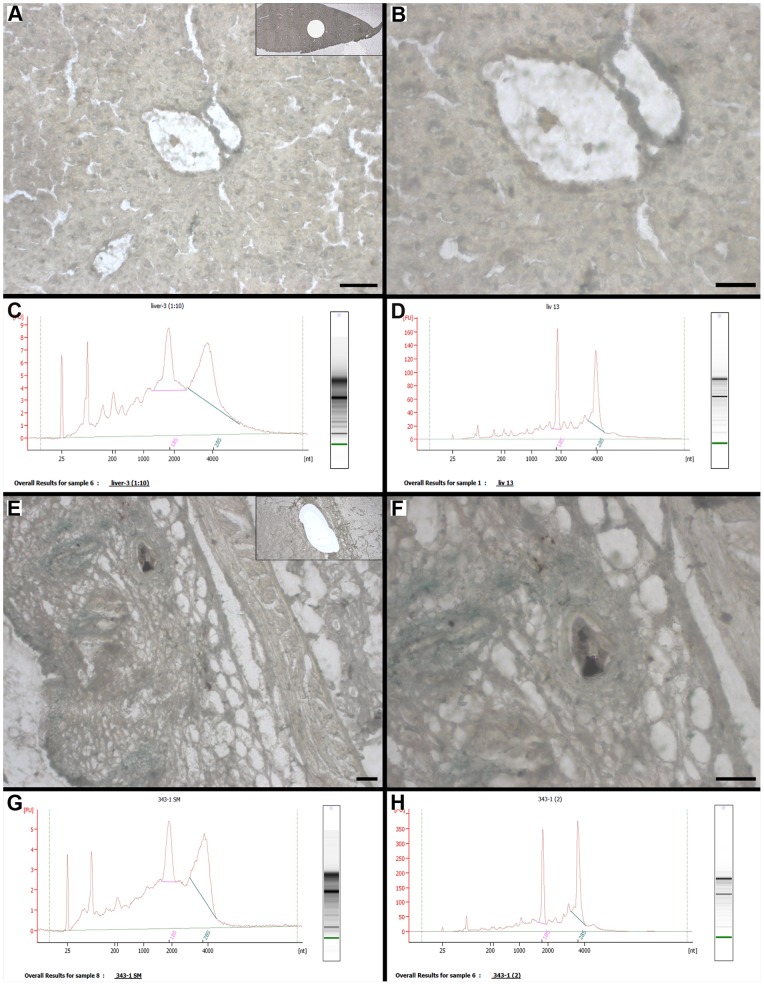
UV-LM on PET-CJ slides (Methyl Green stain), and quality of RNA retrieved from LM targets. (A, B) Mouse liver morphological details on the MMI CellCut Plus dissecting screen at 10x and 40x magnification, respectively: inset shows a circle area of dissected target for RNA retrieval; (C, D) Representative Agilent electropherogram of LM RNA extracted from mouse liver on slides without (contaminant is present in RNA sample) and with pretreatment solution (high quality RNA) before adhesive application, respectively; (E,F) Mouse skin morphological details on the MMI CellCut Plus dissecting screen at 10x and 40x magnification, respectively: inset shows an area of papilloma dissected for RNA retrieval; (G,H) Representative Agilent electropherogram of LM RNA extracted from mouse skin papilloma on slides without (contaminant is present in RNA sample) and with pretreatment solution (high quality RNA), respectively. A, E, F: Scale bars correspond to 100 µm; B: Scale bar corresponds to 50 µm.

Sections of mouse ovary (LM of normal ovarian epithelium) and skin (LM of skin papilloma with a papilloma base) were mounted on glass-CJ slides and dissected on a PixCell® IIe (Arcturus Engineering, Mountain View, CA, USA) with CapSure®HS LCM Cap (Fig. 3A–D). Sections of mouse liver (LM of a one mm diameter circle (Fig. 4A, inset)) and skin (Fig. 4E, inset)) were mounted on PET slides, which were incubated in pretreatment solution and then coated with 15 µl of adhesive. Dissections were performed on a MMI CellCut Plus (MMI Molecular Machines & Industries, Glattbrugg, Switzerland). The dissection time was scaled according to RNA stability in the tissue: 25 minutes-for liver, 20 minutes-for mouse normal ovarian epithelium and 12 minutes-for skin papilloma.

Under a dissecting microscope, the film area of a CapSure®HS LCM Cap containing collected targets was cut out with a razor blade to avoid transferring non-specific debris that typically collects on the cap railing. The cutout was peeled off the cap with Inox #5 forceps (Roboz Surgical Instrument, Co., Dumont, Switzerland) and placed into a 1.5 ml nuclease-free micro-centrifuge tube for high G-force (VWR, West Chester, PA, U.S.A.) (Fig. 3 G–I). The MMI IsolationCap® was used for collection of dissected targets on MMI CellCut Plus. For each LM target, sample collection was conducted minimum in duplicates.

### LM Sample Lysis, RNA Extraction and Quality Assessment

AllPrep® DNA/RNA Micro Kit (Qiagen GmbH, Hilden, Germany) was used for LM sample lysis and RNA purification. For ovary and skin (IR-LM), the dissected material from each of ten slides was lysed individually (35 µl of buffer RLT from the kit). The tubes with samples were vortexed for 30 seconds at maximum speed and incubated for two minutes at RT three times in succession. The films were discarded and lysates were kept on dry ice before transfer to −70°C storage. Before extraction, lysates were thawed on wet ice and combined as a single sample. For liver and skin (UV-LM), dissected targets from a single laser cutting session were transferred to a 1.5 ml tube with 350 µl of RLT buffer, and lysed by vortexing as described above.

RNA was extracted (14 µl elution volume) with the following modifications to the manufacturer’s protocol: (a) 1 µl of diluted (1∶1) linear polyacrylamide (Sigma, Saint Louis, MO, USA) was added to each sample lysate, vortexed for 30 seconds at maximum speed and incubated for 5 minutes at RT before application to the DNA column, (b) the DNA column was discarded after DNA binding, (c) at the RNA binding step the column was centrifuged for 1 minute at 100g following by 2 minute at 16,000 g, (d) at the second washing step the column was incubated for 5 minutes before centrifugation, (e) heated water (+70°C) was used for RNA elution, (f) at the elution step each column was incubated for 5 minutes, centrifuged for 1 minute at 100 g, followed by 1 minute centrifugation at 16,000 g, then re-eluted with 5 minute incubation, followed by a 3 minute centrifugation at 16,000 g.

Quality of LM RNA samples was evaluated with Agilent RNA 6000 Pico Kit on an Agilent 2100 Bioanalyzer (Agilent Technologies, Santa Clara, CA). RNA quality in frozen tissue blocks was evaluated as previously described [4]. Quality assessment of each RNA preparation was carried out minimum in duplicates. The statistical significance was evaluated by two-tailed Student’s t-test with 95% confidence (p≤0.05).

## Results and Discussion

### Cryotomy

For PET-CJ slides we should emphasize several important changes to the CryoJane manufacturer’s cryosectioning manual.

**Figure 5:**
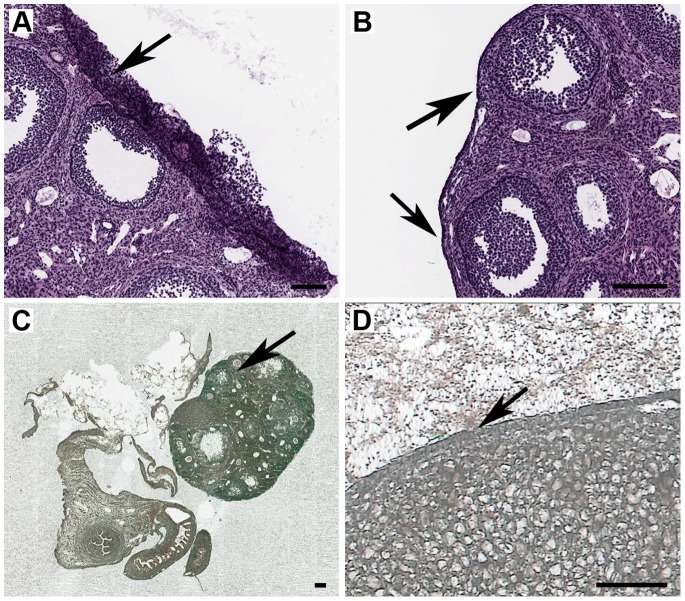
Tissue morphology on routine and glass-CJ slides for IR-LM of mouse normal ovarian epithelium. (A) Routine cryosection of mouse ovary mounted on ++glass slide (H&E stain): arrow indicates ovarian epithelium rolled onto the ovary; (B) CryoJane section of mouse ovary (H&E stain): arrows indicate intact ovarian epithelium; (C) Aperio digital image of scanned non-coverslipped CryoJane section of mouse ovary prepared for IR-LM ((Methyl Green stain): arrow indicates an ovary; (D) PixCell® IIe dissecting screen image of the same section(10x): arrow indicates intact LM target-ovarian epithelium. A–C: Scale bars correspond to 100 µm; D: Scale bar corresponds to 50 µm.

**Figure 6:**
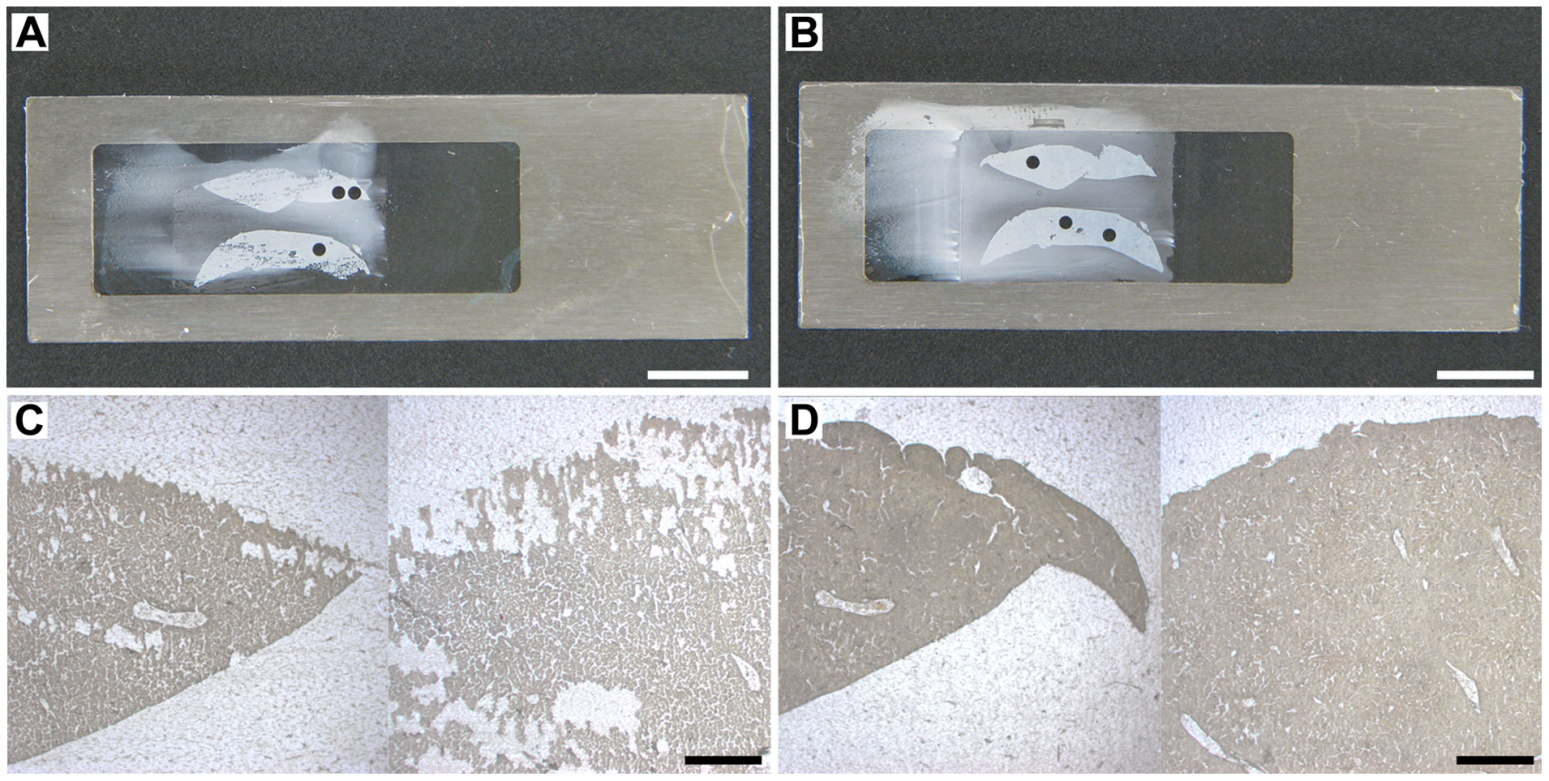
The effect of adhesive volume on the efficiency of CryoJane section transfer to the membrane. (A, B) View of mouse liver CryoJane section (after OCT removal) on membrane slide coated with 10 µl and 15 µl of adhesive, respectively; (C, D) MMI CellCut Plus dissecting screen image (4x) of the same area of a transferred section: incomplete transfer on slides coated with 10 µl of adhesive (C), and complete transfer (D) on slides coated with 15 µl of adhesive. A, B: Scale bars correspond to 1000 µm; C, D: Scale bars correspond to 500 µm.

Firstly, PET-CJ slide should be placed on a MMI SupportSlide, which proved to be invaluable for proper transfer of sections onto membranes during application of the Hand Roller (Leica Microsystems, GmbH, Nussloch, Germany) to the Transfer Adhesive Tape. It effectively prevented the rupture of the fragile membranes under pressure and provided consistent section adherence to the membrane. Section transfer on manually coated PET membranes was complete (Fig. 2 C, F, 3 B, 5 C, 6 B, D) and comparable to glass-CJ slides.

Secondly, PET-CJ slides should be kept at RT before sectioning and cooled down on the UV Flash Unit pad for two minutes before section transfer. Under or overcooling will result in incomplete transfer of sections onto the membrane.

Thirdly, the position of the tape on PET-CJ slide during transfer is crucial for membrane integrity. Mounting transfer tape with a frozen section crosswise to the membrane window didn’t damage the membrane during tape removal on PET-CJ slides (Fig. 2 A, C). In contrast, lengthwise tape application resulted in torn membranes at the spots where the adhesive side of the tape around the section touched the membrane (Fig. 2 B, D).

The OCT around the tissue should overlap the window (Fig. 2A) to keep the laser cutting area of the membrane intact. Damaged membranes affect laser focusing and a laser path, and with significant damage dissection becomes impossible. We recommend embedding LM tissue in a Tissue-Tek® standard disposable Cryomold® 25×20×5 mm (Sakura Finetek, Inc. Torrance, CA, U.S.A.) that matches the size of the “PEEL THIS OFF” area of the Transfer Adhesive Tape (Fig. 1F). The OCT covering adhesive on the tape effectively protected PET membranes from damage during section transfer. Special care should be taken during roller application to the PET-CJ slide positioned on top of the plastic MMI SupportSlide (Fig. 1 G, H).

It proved difficult to obtain a flat section of mouse ovary (Fig. 5A) and skin with intact LM targets by routine cryotomy. In contrast, CryoJane sections of these tissues on ½ × CryoJane slides were flat and provided good morphology suitable for LM (Fig. 3D, 5B). Any tape mounting position is acceptable for section transfer to a glass-CJ slide. Such flexibility in tape orientation facilitates an optimum sample preparation design for complex multi-target LM projects.

It is important to point out that CryoJane also eliminates problems of target loss on PET slides during staining. Poor cryosection adherence to the membrane is common with any type of membrane slides used for LM [15]. Unfortunately, the usefulness of conventional coating solutions for enhanced section adherence to the membrane (such as poly-L-lysine and 3-aminopropyltriethoxysilane) is strongly tissue dependent, with inconsistent results.

### Fixation and Staining

Our staining protocol provided good morphology and contrast on the dissecting screen for ovary (Fig. 3A, 5D) and skin papilloma (Fig. 3B, D) on glass-CJ slides, as well as for liver (Fig. 4A, B) and skin (Fig. 4E, F) on PET-CJ slides.

Application of Vector® Methyl Green (2 ml) to the section after fixation, effectively removed OCT from the tissue section and surrounding adhesive on PET-CJ slides (Fig. 2E). However, on glass-CJ slides the dissolved OCT compound formed deposits on the adhesive and tissue alike when the tissue size was much smaller than the OCT area of the transferred section (Fig. 2G). Trimming OCT blocks to a minimum possible size effectively eliminated OCT from the transferred areas (Fig. 2F). Vector® Methyl Green is our preferred LM stain for the following reasons: (a) staining and OCT removal occur in one step; (b) good contrast on LM dissecting screens of PixCell® IIe and MMI CellCut Plus; (c) it doesn’t deplete RNA yield in LM samples, compared with other LM suitable stains [16]–[18]; (d) it doesn’t modify RNA [16]–[18]; (e) contrast on the LM screen can be enhanced by counterstaining with eosin Y diluted (1∶6 or 1∶10) in 100% ethanol. Our second stain of choice is “One-step Cresyl Violet Acetate/Eosin Y” [4], which can be also used in combination with Vector® Methyl Green with the following modification to the reagent mixture: 200 µl of cresyl violet stock solution, 100 µl of eosin Y, and 400 µl of RNAse-free water.

### Coating of PET Slides with CryoJane Solutions

After incubation of PET slides in pretreatment solution containing acetone, we observed minute patches of membrane detachment from the metal frame along the edges but it didn’t interfere with the CryoJane cryotomy or LM. We assume that this method could be successfully used with the PEN slides (glass membrane slides) as well, since the membrane adhesive was not significantly compromised by solvent.

However, omission of slide pretreatment with solution A before adhesive application negatively affected quality of RNA extracted from both liver and skin targets on PET-CJ slides. On Agilent electropherograms the ribosomal peaks were not completely separated, the baseline was significantly elevated, and numerous bands were present prior to the ribosomal bands (Fig. 4C, G). In contrast, a pattern of high quality RNA was observed for targets on PET slides incubated in pretreatment solution before adhesive application (Fig. 4D, H). Also, the RNA integrity numbers (RIN) were lower for both liver and skin on slides without pretreatment compared to slides pretreated before adhesive application (Table 1, p≤0.05). Comparison of RNA electropherograms and corresponding e-gel images suggested the presence of a contaminant (possibly adhesive) that co-purified with RNA in samples without pretreatment.

**Table 1:** Effect of PET-CJ slide coating conditions on RNA integrity of LM targets.

Target tissue	Pretreatment with Solution A	Volume of Solution B (µl)	Mean RIN*±SD** (n = 4)
Mouse liver	no	20	6.4±0.5
Mouse liver	no	30	6.3±0.2
Mouse liver	no	50	5.5±1.2
Mouse skin	no	30	6.1±0.1
Mouse skin	no	50	5.5±1.3
Mouse liver	yes	10	nd
Mouse liver	yes	15	7.1±0.2
Mouse liver	yes	30	7.2±0.3
Mouse liver	yes	35	7.1±0.1
Mouse skin	yes	30	7.2±0.3
Mouse skin	yes	50	7.2±0.2

* RIN = RNA Integrity Number;

** SD = standard deviation; nd = no data.

The applied volume of adhesive didn’t affect RNA quality regardless of pretreatment. For liver, differences were insignificant between average RINs for 20, 30 and 50 µl of adhesive applied to the membrane without pretreatment as well as for 15, 30 and 35 µl of adhesive on pretreated membrane. The same was true for skin sections on slides coated with 30 and 50 µl of adhesive with or without pretreatment (Table 1; p≤0.05).

The flexibility with the adhesive volume allows preparation of PET-CJ slides of desired strength (as commercial glass-CJ slides) to facilitate cryosectioning of different types of tissue. We have noticed that adipose tissue of human breast biopsy remains intact on PET slides manually coated with 15 µl of adhesive compared to significantly damaged tissue on ½ × glass-CJ slides (unpublished data).

However, section transfer was affected by the volume of adhesive applied to the PET slide. A good transfer of sections from tape to the membrane was achieved with all tested volumes of adhesive except 10 µl when incomplete sections were observed after mounting (Fig. 6A–D).

Laser focusing and the efficiency of laser cutting were also affected by the adhesive volume. On 10 and 15 µl slides, laser parameters and reproducibility of cutting efficiency were similar to control PET slides without CryoJane coating. Targets on 20 µl slides were cut effectively with a 10 unit increase of laser power required for PET control slides. Complete cutting on 30 and 35 µl slides was achieved with laser power increase of 15 and 25 units, respectively, but on 50 µl slides not all the targets were completely cut out even with maximum (100%) laser power.

The thick layer of adhesive (30–50 µl) affected the laser path; the laser couldn’t connect the start and end point of the target annotation line during cutting. Closing a drawing on the dissecting screen with a crossing line beyond the start point allowed for a complete cutting of an annotated target. With these coating conditions, large targets (≥500 µm in diameter) were too heavy to lift with a MMI IsolationCap®. However, such dissected targets were easily picked up by fine tip forceps directly from the slide on the microscope dissecting stage.

Several points should be addressed for the coating procedure. The container of adhesive should be covered at all time and application should be rapid to avoid adhesive thickening. A drop of adhesive should be positioned at the beginning of the membrane window and spread only in the window area to avoid contamination of the metal frame. Contaminated metal frames have the potential to stick to the CryoJane UV Flash Unit pad or a gloved hand causing damage of the fragile membrane during detachment. For the same reasons, we recommend storing PET-CJ slides in a slide box without covering the adhesive side with thin protective plastic present on the commercial slide. At the start of the coating movement it is important to allow a drop of adhesive to spread evenly beneath the plastic coverslip along the edge of the slide window. This provides for consistent thickness of the adhesive on the membrane which in turn facilitates laser focusing and cutting efficiency.

Based on results the best coating method is incubation of PET slides in pretreatment solution before application of 15 µl of adhesive. Four month storage of pretreated PET slides in a slide box at RT before adhesive application didn’t affect the efficiency of section transfer to the membrane.

### Laser Microdissection

CryoJane LM preparation proved suitable for successful LM on both glass-CJ and PET-CJ slides. Additional drying of LM slide in a desiccator allowed to decrease power required for efficient film melting by PixCell® IIe laser and membrane cutting by MMI CellCut Plus laser. This also improved laser focusing.

Consistent laser focusing throughout the cutting path and complete target pick-up by MMI IsolationCap® on PET-CJ slides was observed for both liver and skin. During LM the performance of PET-CJ slides coated with 15 µl was not altered by the adhesive, and they required the same laser cutting parameters as regular PET slides.

Nonspecific contamination of collected LM targets is an intrinsic problem with Arcturus CapSure® and HS® LCM Cap technology [2], [4]–[6]. Glass-CJ slides dramatically decreased nonspecific target contamination from tissue adjacent to a melted spot on the cap (Fig. 3A–D). However, some contamination was observed on the cap railing. We successfully avoided this contamination by cutting the film around captured tissue and peeling the film with the target off the cap. We prefer this method to cleaning aids (cleaning pads, sticky note pads) which deplete captured LM target during the removal of non-specific material [4]. Moreover, the peeled off target can be incubated in a regular 1.5 ml tube with a variable volume of lysis buffer. Thus, there is no risk of sample loss during incubation due to the leakage of HS Cap assembly with Arcturus ExtracSure™Device.

Regardless of tissue type (skin or ovary), targets embedded in the film on HS Caps were completely detached from glass-CJ slides, leaving the adhesive on a slide (Fig. 3A, D). This was true for human breast ductal epithelium, stromal and adipose tissue, and immuno-labeled human melanoma cell pellet (data not published). Thus, the bonding strength of polymerized adhesive to the section is not tissue specific, and the use of glass-CJ slides helped to overcome a well-known problem of tissue type dependent adherence between glass slide and section impairing LM dissections [2], [4], [5]. It also eliminated the need of testing for efficient target pick-up on different types of slides prior to cryotomy.

CryoJane two-step coating didn’t affect RNA integrity in LM samples (mouse liver); the difference between average RIN numbers for LM targets from PET-CJ and PET slides was not significant (Table 2; p≤0.05).

**Table 2:** Effect of LM sample preparation on RNA integrity of LM targets.

Tissue target	Mounted section	Stained section	Slide type	IR LM	UV LM	Mean RIN* ± SD** (n = 4–12)
Mouse liver	no	no	none	none	none	9.0±0.2
Mouse skin	no	no	none	none	none	9.3±0.3
Mouse ovary	no	no	none	none	none	9.4±0.2
Mouse liver	yes	yes	PET-CJ	no	yes	7.1±0.2
Mouse liver	yes	yes	PET	no	yes	7.3±1.2
Mouse skin	yes	yes	PET-CJ	no	yes	7.2±0.2
Mouse skin	yes	yes	Glass-CJ	yes	no	7.9±0.3
Mouse ovary	yes	yes	Glass-CJ	yes	no	8.2±0.1

* RIN = RNA Integrity Number;

** SD = standard deviation.

Our fixation, staining protocol and dissection time frame allowed high quality RNA retrieval from LM samples. RNA quality in ovarian, skin and liver samples (average RINs of 8.2, 7.2 and 7.1, respectively) (Fig. 3E, F, 4D, H) is considered suitable for RNA amplification and wide range of downstream molecular analyses [5], [19], [20].

We have observed some RNA degradation through cryotomy, staining and dissection on both glass-CJ and PET-CJ slides, which is consistent with the reports from different LM studies [2], [5], [7], [8], [14], [16], [17]. On glass-CJ slides, average RIN for ovarian and skin LM sample decreased by one unit compared to the RIN in OCT tissue blocks. On PET-CJ slides, RIN for liver and skin LM sample decreased by two units compared to the RIN in OCT tissue blocks (Table 2; p≤0.05). Statistical comparison of RINs in skin LM samples obtained from glass-CJ and PET CJ slides showed that sample preparation and dissection on PET-CJ slides caused more damage to target RNA than on glass-CJ slide (Table 2; p≤0.05). We suspect that the UV laser used for the dissection of tissue on PET-CJ slides impacted RNA more strongly than the IR laser used for glass-CJ slides.

### Conclusions

The CryoJane Tape-Transfer System® was found suitable for laser cutting (UV-LM) microdissection technology. The CryoJane coating and section transfer procedure we have established for membrane slides allowed for effective section transfer, laser cutting and target collection for different types of tissue of different RNA stability. Our protocol resulted in retrieval of high quality LM RNA. This marks the first assessment of CryoJane application to membrane LM slides for laser cutting projects.

In addition to the previously established advantage in obtaining complete frozen sections, use of CryoJane on glass slides greatly reduced the contamination of the target with adjacent non-specific tissue during LM and provided complete target detachment from the slide.

We have established a standard fixation and staining protocol suitable for both glass-CJ (IR-LM) and PET-CJ (UV-LM) slides. It preserved RNA in LM targets and provided adequate morphology and good contrast on the LM dissecting screen. Considering the damaging effect of UV lasers on nucleic acids [2], [6], a combination of different lasers and slides could be beneficial for multi-target LM projects. Glass CJ slides should be used for RNA retrieval from small targets (≤30–70 µm in diameter) by IR-LM, and PET-CJ slides for larger targets by UV-LM. By providing a foolproof cryosectioning procedure for any type of tissue, maximizing the use of unique samples and making complex LM projects feasible, the CryoJane can be the foundation of a standard LM slide preparation protocol. It will expand the potential of laser microdissection as a valuable research tool in a wide range of large-scale animal and human molecular pathology studies.
